# Deep high-temperature hydrothermal circulation in a detachment faulting system on the ultra-slow spreading ridge

**DOI:** 10.1038/s41467-020-15062-w

**Published:** 2020-03-10

**Authors:** Chunhui Tao, W. E. Seyfried, R. P. Lowell, Yunlong Liu, Jin Liang, Zhikui Guo, Kang Ding, Huatian Zhang, Jia Liu, Lei Qiu, Igor Egorov, Shili Liao, Minghui Zhao, Jianping Zhou, Xianming Deng, Huaiming Li, Hanchuang Wang, Wei Cai, Guoyin Zhang, Hongwei Zhou, Jian Lin, Wei Li

**Affiliations:** 1 Key Laboratory of Submarine Geosciences, MNR, Second Institute of Oceanography, MNR, 310012 Hangzhou, China; 2 School of Oceanography, Shanghai Jiao Tong University, 200240 Shanghai, China; 3 Department of Earth Sciences, University of Minnesota, Minneapolis, MN 55455 USA; 4 Department of Geosciences, Virginia Polytechnic and State University, Blacksburg, VA 42061 USA; 5 College of Geoexploration Science and Technology, Jilin University, 130026 Changchun, China; 6 Institute of Geophysics and Geomatics, China University of Geosciences, 430074 Wuhan, Hubei China; 7 Institute of Deep-Sea Science and Engineering, Chinese Academy of Sciences, 572000 Sanya, China; 8 Department of Geophysics, School of Earth & Space Sciences, Peking University, 100871 Beijing, China; 9 The Federal State Budgetary Institution, Academician I.S. Gramberg All-Russia Scientific Research Institute for Geology and Mineral Resources of the Ocean, Saint-Petersburg, 190121 Russia; 10 Key Laboratory of Ocean and Marginal Sea Geology, South China Sea Institute of Oceanology, Chinese Academy of Sciences, 510301 Guangzhou, China; 11 Department of Geology and Geophysics, Woods Hole Oceanographic Institution, Woods Hole, MA 02543 USA

**Keywords:** Solid Earth sciences, Geochemistry, Geology

## Abstract

Coupled magmatic and tectonic activity plays an important role in high-temperature hydrothermal circulation at mid-ocean ridges. The circulation patterns for such systems have been elucidated by microearthquakes and geochemical data over a broad spectrum of spreading rates, but such data have not been generally available for ultra-slow spreading ridges. Here we report new geophysical and fluid geochemical data for high-temperature active hydrothermal venting at Dragon Horn area (49.7°E) on the Southwest Indian Ridge. Twin detachment faults penetrating to the depth of 13 ± 2 km below the seafloor were identified based on the microearthquakes. The geochemical composition of the hydrothermal fluids suggests a long reaction path involving both mafic and ultramafic lithologies. Combined with numerical simulations, our results demonstrate that these hydrothermal fluids could circulate ~ 6 km deeper than the Moho boundary and to much greater depths than those at Trans-Atlantic Geotraverse and Logachev-1 hydrothermal fields on the Mid-Atlantic Ridge.

## Abstract (web summary)

Magmatic and tectonic activity at mid-oceanic ridges can give detailed insights into high-temperature hydrothermal circulation of fluids. The authors here present geochemical and geophysical datasets that suggest a hydrothermal system penetrating the upper lithospheric mantle at an ultra-slow spreading mid-oceanic ridge.

## Introduction

Slow (1.2– 5.5 cm year^−1^) and ultra-slow (<1.2 cm year^−1^ full spreading rate) spreading ridges are typically characterized by low melt production^1^. Conductive cooling limits melt production and the mantle rocks are often tectonically exposed onto the seafloors^1–4^. This distinctive geological setting allows faulting to be particularly extensive and to penetrate deep into the crust and upper mantle, thereby potentially forming pathways for enhanced hydrothermal fluid circulation^5^. The thermal structure of a spreading center is controlled in part by this hydrothermal circulation that can result in the formation of large polymetallic sulfide deposits^6^. The depth of the hydrothermal circulation is important in the efficiency of lithospheric cooling^7^, the style of accretion of the lower oceanic crust^8^, and the potential to leach ore-forming elements from the rock^6^. The depths of hydrothermal circulation on detachment faults at oceanic core complexes (OCC) of slow spreading ridges, such as Trans-Atlantic Geotraverse (TAG) and Logatchev-1 hydrothermal fields, can be traced up to ~7 and ~6 km below the seafloor (bsf), respectively^9,10^. The chlorine-excess in melt inclusions hosted by basaltic rocks in the South Mid-Atlantic Ridge (MAR) and Gakkel Ridge offers further geochemical clues that hydrothermal alteration reaches lower crustal depths^11^. Despite these observations, however, the origin and depth of the hydrothermal fluids in active high-temperature vent fields at the ultra-slow spreading ridge has not, until now, be investigated in any detail.

**Fig. 1:**
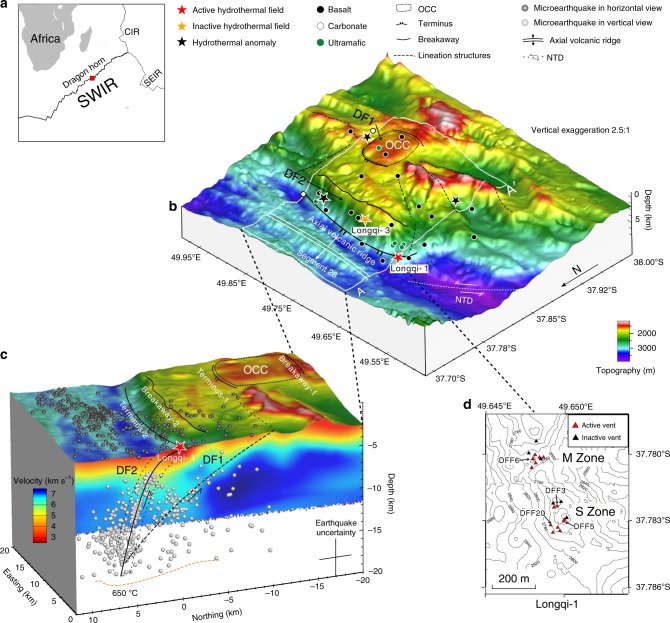
Location and sketch of the Dragon Horn area. **a** Location of the Dragon Horn area at 49.7°E. **b** Bathymetry with tectonic and hydrothermal features. The corrugated surface of the OCC extends about 5 km × 5 km to the south in a series of steep scarps typical of breakaways, and to the north ending at the axial valley wall. NTD represent non-transform discontinuities. Location of hydrothermal fields and anomaly sites are shown as colored stars, including active Longqi-1 hydrothermal field (red), inactive Longqi-3 field (orange), and hydrothermal anomalies (black). **c** Three-dimensional view of the detachment fault zone. It shows the distribution of the epicenters, located within the white rectangle in Fig. 1b. The AA′ seismic velocity profile comes from a wide-angle seismic experiment^15^. **d** High-resolution bathymetry and distribution of vents in Longqi-1 field. The bathymetry data were acquired by near-bottom underwater vehicles, human-occupied and autonomous underwater vehicles. DFF3, DFF5, DFF6, and DFF20 represent the vents from which the hydrothermal fluids were sampled in this paper.

The Longqi-1 hydrothermal vent field (~49.7°E) in the Dragon Horn region (Fig. 1) of the ultra-slow spreading Southwest Indian Ridge (SWIR) exhibits high-temperature hydrothermal vents associated with a major detachment fault system and has been the subject of recent intensive studies^12^, providing an opportunity to examine this problem. Here, we show the comprehensive geophysical and geochemical investigations on this hydrothermal field, which are based on the ocean bottom seismometers (OBS), the Jiaolong human-occupied vehicle, and Qianlong II autonomous underwater vehicle. A concerted effort was also undertaken to track the pathway of hydrothermal circulation using a two-dimensional (2D) numerical model of circulation in a NaCl-H_2_O fluid system to verify whether the hydrothermal fluids derived from the depth could circulate up to the seafloor and vent at the observed high temperatures. Our results show that the hydrothermal circulation below the Longqi-1 field is associated with a detachment system penetrating to the depth of 13 ± 2 km below the seafloor, and the hydrothermal fluids could circulate ~6 km deeper than the Moho boundary that is much deeper than those at TAG and Logachev-1 hydrothermal fields on the MAR.

## Results

### Geological setting

The Dragon Horn area is located on the south flank of the SWIR segment 28 (~49.7˚E), following the nomenclature described previously^13^. The corrugated surface of the detachment fault of the OCC exposed on the southern ridge flank covers an area of approximately 5 km × 5 km (Fig. 1b). The basalt-hosted Longqi-1 active vent field is located at the edge of this OCC at water depths of 2700–2900 m on the southwest wall of the axial rift valley and comprises two sulfide-bearing vent zones named S zone and M zone, respectively (Fig. 1b, d)^12^. The highest measured fluid temperature of 379 °C is associated with the DFF6 (Dragon Flag Field: English name of the Longqi-1 Field) vent site of the M zone (Fig. 1d, Supplementary Table 1). The active Longqi-1 vent field together with the inactive Longqi-3 hydrothermal field and a hydrothermal plume anomaly site form a possible linear mineralized zone along terminus 2 of the detachment fault 2 (DF2) (Fig. 1b, c), where serpentinized peridotite outcrops have been observed and sampled. Additionally, consolidated carbonate sediments representing low-temperature hydrothermal processes were also found on the east side of the OCC (Fig. 1b)^14^.

### Seismic observation

We located 512 microearthquakes with local magnitudes of −0.5 < M_L_ < 3 by a double-difference relocation method during a series of discontinuous OBS monitoring experiments. The relocated hypocenters were divided into two sections near the non-transform discontinuities (NTDs) and OCC, respectively (Supplementary Fig. 1). Seismic activity on the west side of Longqi-1 is attributed to a network of brittle faults and fractures in the NTD zone. Therefore, only hypocenters in the OCC zone, which are associated with detachment faulting beneath the southern flank of the axial valley (within white rectangle in Fig. 1b), are considered in this study. Most events are located along the axial volcanic ridge at a depth of 13 ± 2 km bsf and shoale to ~3 km bsf to the south. The cross-sectional view suggests that most of the hypocenters are focused along two seismically active structures within 1 km offset (inside of dotted curve in Fig. 1c), with some of the earthquakes occurring outside the two seismic zones.

We infer that the earthquakes beneath Longqi-1 field are more possible induced by tectonic activities rather than by magmatic intrusions, based on the following observations. Firstly, active-source wide-angle seismic data conducted along the SWIR from 49.3˚E to 50.8˚E^15,16^ clearly shows that there is no crustal magma chamber beneath Longqi-1 hydrothermal field and that no volcanic tremors were recorded by the OBS network. Secondly, dike-induced earthquakes associated with magma intrusions typically show hypocenters migrating with time along a narrow path^17^; however, the distributions of the earthquakes in our case show no migration behavior with time. In addition, the similar spatial distribution of earthquakes has been observed at Logatchev-1, TAG, and Irinovskoe hydrothermal fields at the MAR, which were explained as the response to the extension of the detachment faults^9,10,18^. The occurrence of scattered microearthquakes outside the regions where a majority of hypocenters are located has also been observed in previous studies at TAG^9^ and 13˚20′N on the MAR^18^. These scattered earthquakes are typically inferred to be due to the volume expansion accompanying serpentinization^19^.

Overall, we define two detachment faults (DF1 and DF2 in Fig. 1c) in the Dragon Horn area based on the morphology, geological sampling, and seismic data. First, we have documented that the smooth seafloor is a detachment fault by sampling of exhumed mantle-derived peridotite (Fig. 1b). Second, most of recorded microearthquake epicenters project along the trace of two detachment fault zones (Fig. 1c). Finally, a three-dimensional P-wave seismic velocity model shows that the footwall of the detachment faults beneath the Dragon Horn area is characterized by shallow, high crustal velocities and a strong vertical velocity gradient (Fig. 1c, Supplementary Fig. 2).

### Fluid chemistry

**Fig. 2:**
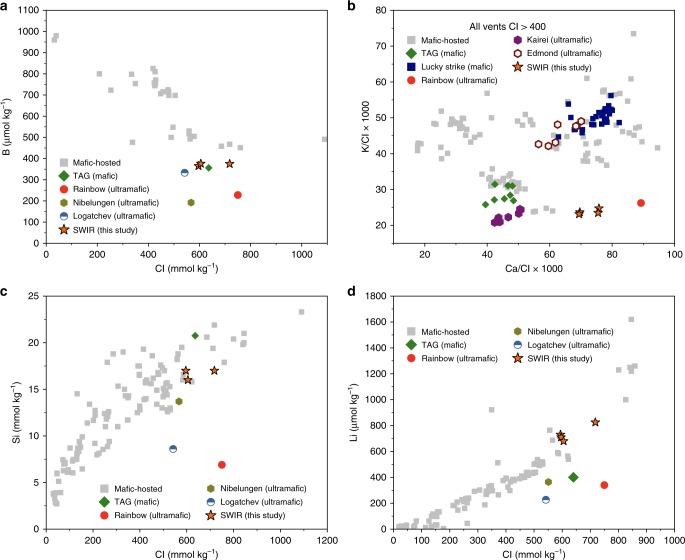
Longqi-1 hydrothermal vent fluids in comparison with vent fluid compositions from other vent sites. **a** Dissolved B versus chloride; **b** dissolved K/Cl versus Ca/Cl; **c** dissolved silicon versus chloride; **d** dissolved lithium versus chloride (see text). Data sources for mafic-hosted systems are as follows: EPR^57–61^; JdFR: ASHES^62^; Southern JdFR^63^; MEF^28,64^; MAR: TAG^43,65,66^; and Lucky Strike^30^. Other host lithologies on MAR: Rainbow^67^; Nibelungen (8˚18′S)^29^; Logatchev (14˚45′N)^38^; and Central Indian Ridge^68^ are displayed for comparison. All the error bars are smaller than the scale of the symbols.

The volatile gas and element concentrations and O–H isotopic compositions of the Longqi-1 venting hydrothermal fluids are shown in the Supplementary Tables 1 and 2. The boron (B), potassium (K), chlorine (Cl), silicon (Si), and lithium (Li) concentrations are compared with those of venting fluids from other vent sites along the global mid-ocean ridges (Fig. 2). Compared with vent fields hosted by mafic rocks, the Longqi-1 vent fluids show obvious depletion in dissolved boron (Fig. 2a) and lower K/Cl ratios (Fig. 2b), while their Si and Li concentrations are within the typical range of global mafic rock-hosted vent fluids (Fig. 2c, d). The concentrations of H_2_, CH_4_, and H_2_S in the high-temperature vent fluids are considerably higher than those observed at TAG (Supplementary Table 1), a detachment-related hydrothermal system at 26°N on the MAR. The δ^18^O and δD (δ^18^O (D) [‰] = (*R*_sample_/*R*_reference_−*R*_reference_) × 1000, where *R* is the ratio of ^18^O/^16^O or D/H, reference is the Vienna Standard Mean Ocean Water (VSMOW)) of the vent fluids vary from 0.18‰ to 1.21‰, and from 0.8‰ to 3.9‰, respectively, both of which are considerably higher than that of local bottom seawater (Supplementary Table 2).

## Discussion

The distribution of microearthquakes along the DF2 is similar to that observed at TAG on the MAR; however, our results demonstrate that DF2 penetrates ~6 km deeper than the detachment fault at TAG as inferred from seismic data^9^ (~13 ± 2 vs. ~7 ± 1.1 km) (Fig. 1c). Although some microearthquakes at ultra-slow ridges have been detected deeper than at the Dragon Horn area, e.g., 16 km bsf for 85°E/85°N Gakkel Ridge^20^ and 17 km bsf for 12.5°~14.5°E SWIR^21,22^, no high-temperature hydrothermal activities were found along these ridges. The maximum depth of microearthquakes in these regions reflect the variation in crustal thickness along-axis because of poor and discontinuous melt supply at these ultra-slow spreading centers^22^. The steep and deep DF2 with associated serpentinized peridotites exposed at the seafloor may be at an initial stage in the rolling-hinge model, in which the oceanic detachment faults initiate at high dips and rotate to low-angle geometries as displacement increases^23^. In contrast, DF1 with scattered epicenters has a much lower angle, as indicated by flexural exhumation of the OCC (dashed line delineated area in Fig. 1b) and the dome-shaped surface. Following the rolling-hinge model and based on the lower angle of the fault, it is believed that DF1 is more mature than DF2 (ref. ^23^). These two detachment faults jointly compose a twin detachment faulting system (Fig. 1c). The detachment faults appear to provide the main circulation path for the hydrothermal fluids^9,10,15,24^. An important consequence of this geometry is that deep detachment faults would allow the seawater to penetrate to a great depth along it and a long fluid circulation/reaction path.

The relatively long circulation/reaction path for Longqi-1 hydrothermal fluids is supported by the geochemical data. The depletion in dissolved boron in the vent fluid from Longqi-1 hydrothermal field (Fig. 2a) suggests seawater reaction with ultramafic rocks, where experimental data have documented the tendency of boron to partition into serpentine and/or chlorite from solution at a range of temperatures and pressures (200–300 °C at pressure around 500 bars)^25–27^. As with TAG vent fluids (Fig. 2a), boron removal could occur during recharge or during more deeply seated reaction where increasingly higher temperatures and lower fluid/rock mass ratios would prevail. Seawater (usually with Cl of 550 mmol kg^−1^, and B of 420 μmol kg^−1^ (ref. ^28^)) passage through this region of the crust would not only lead to boron removal from the fluid, but the continued hydrolysis and reaction of olivine and plagioclase could contribute to the observed elevated dissolved chloride concentrations^27,29^. Chloride concentrations in vent fluids elevated relative to seawater is a noteworthy feature of many detachment-related hydrothermal systems at mid-ocean ridges^30–32^. Although moderately low and high dissolved chloride concentrations in vent fluids are often accounted for by phase separation effects in the NaCl–H_2_O system, especially in basalt-hosted hydrothermal systems with well-defined shallow magma chambers (e.g., East Pacific Rise 9°N), the same is less likely here owing to the high hydrostatic pressures that prevail at the inferred depths of the SWIR vent system. The preponderance of olivine and plagioclase bearing plutonic rocks, needed for hydrolysis reactions, is also suggested by the usually low K/Cl, high Ca/Cl, and moderately high dissolved methane of Longqi-1 vent fluids (Fig. 2b, Supplementary Table 1). Entrapment of mantle-derived CO_2_ and its reduction to methane in fluid inclusions in plutonic rocks of the ocean crust at the SWIR has been long recognized^33–36^ and subsequent leaching by hydrothermal alteration^37^ could account for the methane dissolved in the vent fluids reported here.

Consistent with hydrothermal vent fluids from the slow spreading MAR, hydrogen and oxygen isotope data for Longqi-1 vent fluids (δ^18^O from 0.18‰ to 1.21‰, δD from 0.8‰ to 3.4‰, Supplementary Table 2) are distinctly elevated and indicate long reaction paths accompanied by intensive water–rock interaction as isotopic fractionation increases with decreasing water/rock ratios, even at moderately high temperatures^32,38–42^. The moderately high dissolved Si and Li concentrations (Fig. 2c, d; Supplementary Table 1), however, are not consistent with serpentinization, although these data could be explained by subsequent passage of the evolved seawater through basaltic rocks, as is likely the case at TAG, where near seafloor tectonic processes inherent to detachment faulting systems make this possible^36,37^. Cs and Rb concentrations are also similar between the TAG and Longqi-1 vent fluids (Supplementary Table 1). These data, together with the low dissolved H_2_ (Supplementary Table 1), suggest phase equilibria involving plagioclase, chlorite, and perhaps epidote solid solutions ± quartz^31^. These water–rock reactions would help to account for the relatively low measured pH (~3.15–3.6) and high Fe and Ca, especially considering the relatively high dissolved chloride concentrations (Supplementary Table 1, Fig. 2d), which can enhance the solubility of these elements^35,43^. In effect, a reasonable model for the chemical composition of the high-temperature Longqi-1 vent fluids is somewhat analogous to hybrid models (both mafic and ultramafic rocks are involved in the water–rock interaction zone) advanced earlier to account for vent fluid chemistry associated with regions of the Kairei^44^ and the Nibelungen^29^ hydrothermal fields. This is consistent with constraints imposed by the existence of deeply penetrating and high permeability faults that focus fluid flow and allow reaction with heterogeneous crustal components inherent to detachment fault systems. As shown by the velocity structure (Fig. 1c), the thickness of the mafic oceanic crust beneath the Longqi-1 hydrothermal field would be 3–6 km (7.3 km isovelocity contour16 in the Supplementary Fig. 2). This means that the fluid-chemical signatures of the ultramafic rock could not be from shallow depth. Thus, the reaction path of fluids with the hybrid mafic and ultramafic rocks should be long and derive from the depth.

**Fig. 3:**
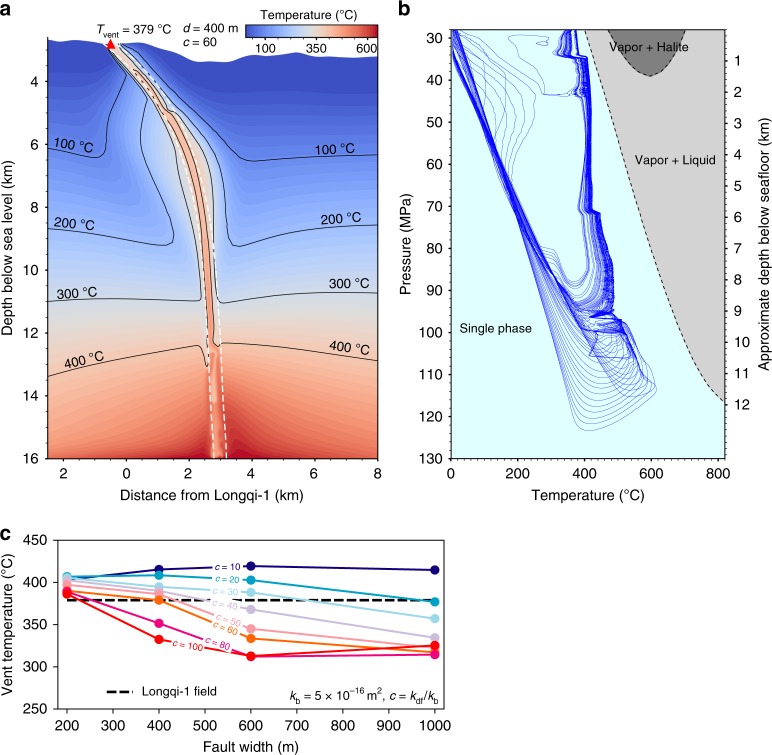
2D numerical model of hydrothermal circulation. **a** An example hydrothermal modeling with constant fault width and the permeability contrast between fault and background. In this case, the assumed fault width (*d*) is 400 m, and the permeability contrast (*c* = *k*_df_/*k*_b_) is 60 (*k*_df_ = 3 × 10^−14^ m^2^, *k*_b_ = 5 × 10^−16^ m^2^). Temperature field distribution is at quasi-steady state, and the isotherms for 100, 200, 300, and 400 °C are shown in green and the simulated vent field with high temperature of 379 °C is shown by red triangle. **b** The temperature–pressure paths of fluid tracers in our 2D numerical model. The blue lines are the modeled possible circulation paths for the hydrothermal circulations. Dashed lines mark phase regions (filled in different colors) of seawater. **c** The comparison of the assumed fault width with the predicted venting temperature. *c* = *k*_df_/*k*_b_ = 10–100, and the horizontal dashed line represent the observed highest temperature of Longqi-1 venting fluids.

For a hydrothermal system associated with steep and deeply penetrating detachment faults and a considerably long reaction path, the location of the underlying heat source is of particular interest. The highest measured temperature of the venting fluid of the Longq-1 hydrothermal fluids is as high as 379 °C, which, combined with an estimated heat output of ~250 ± 100 MW^45^, requires a magmatic heat source^46^. The seismic velocity structures and the distribution of the microearthquakes preclude the existence of a crustal magma chamber or gabbro intrusion beneath the Longqi-1 field shallower than 13 ± 2 km bsf (Fig. 1c). Therefore, the heat source would most likely be located below the depth of 13 ± 2 km and presumably under the axial volcanic ridge. To investigate the dynamics of the hydrothermal circulation and whether the discharge of high-temperature fluids at Longqi-1 can be derived from a very deep magmatic heat source through such a deep and steeply dipping fault zone, we conducted numerical simulations using a hydrothermal model solving for porous flow of seawater^24,47^ (see Methods for the details and Supplementary Fig. 3 for the setting of initial conditions). This numerical modeling allows us to investigate the temperature of the venting fluids at the seafloor for a range of given initial conditions including the width of the fault zone and the permeability contrast between the fault zone and the background (here the background is the oceanic lithosphere without fault zone) (*k*_df_/*k*_b_). In the modeling, we set the heat source depth at the bottom of the lower limit of the microearthquakes (~13 km bsf), and the temperature to 650 °C, which is consistent with the isotherm deduced from the brittle–ductile transition of the lithospheric mantle inferred from the maximum depth of the microearthquakes^22,48,49^. The results show several important clues to the Longqi-1 hydrothermal circulation. As shown in Fig. 3c, for a fault zone with a width around 400 m, the temperature of the venting fluids varies from ~380 to ~410 °C when the permeability of the fault zone varies from 10 to 60 times the background. For a fault zone with relatively low permeability (*k*_df_/*k*_b_ < 30), the temperature of the venting fluid is higher than 350 °C but is not so sensitive to the width of the fault zone. For a wide range of fault zone width (200–1000 m) and a wide range of *k*_df_/*k*_b_ (10–100), the predicted temperature of the venting fluids is greater than 300 °C (Fig. 3c, Supplementary Fig. 4). When the *k*_df_/*k*_b_ is set as 60, which sets the permeability of the fault zone close to the estimated permeability of the discharge zone (3 × 10^−14^ m^2^), based on the heat output (see Methods for details), a fault zone width of ~200–400 m will allow the venting fluids to maintain a temperature consistent with the observed value (379 °C) (Fig. 3a). This response of vent temperature to permeability contrast and fault width is similar to that reported by ref. ^24^, while the simulated discharge mass flux (10^−4^–5 × 10^−4^ kg m^−2^ s^−1^) is also in the same range as reported by refs. ^24,47^. Pressure–temperature paths of numerical fluid tracers (Fig. 3b) further suggest that venting fluids had reached depths of up to 13 km bsf and that the fluid always remained in the single phase region of seawater. Both findings are very different from modeling results for the fast-spreading East Pacific Rise^47^, where fluids circulate no deeper than ~7 km bsf and pressure–temperature paths do intersect with seawater phase boundaries. This is also consistent with inferences based on the fluid geochemistry as discussed above. We also conducted a set of numerical models to test the response of vent temperature to the permeability contrast of DF1 and DF2 (*k*_df2_/*k*_df1_) (Supplementary Fig. 5). The results show that if the permeability of DF1 is higher than the background permeability, DF1 becomes the pathway for recharge flow toward the foot of DF2, but hot rising fluids never flow through DF1. This is in agreement with the absence of high-temperature hydrothermal activity near the terminus of DF1. The observations that there is no obvious signature of hydrothermal circulation near the terminus of DF1, and that there are considerably fewer microearthquakes along DF1 compared with DF2 (Fig. 1c), supports the interpretation that DF1 has much lower permeability than DF2 (high *k*_df2_/*k*_df1_). Thus, the simulation results support the idea that the high-temperature hydrothermal fluids at Longqi-1 originate from as deep as ~13 ± 2 km bsf, given that the temperature of the heat source could be up to 650 °C.

**Fig. 4:**
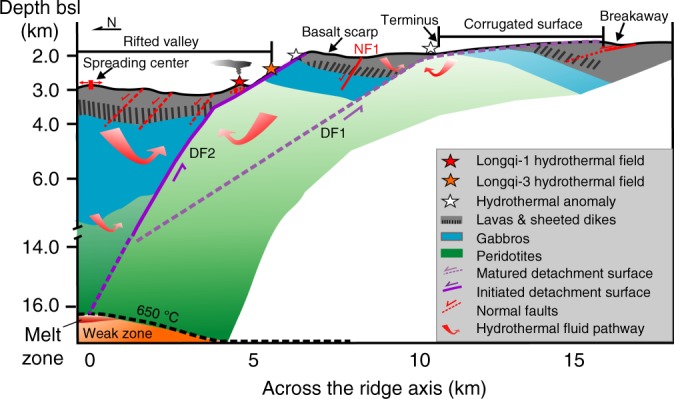
Proposed geological model for hydrothermal circulation of the Dragon Horn area. The tectonic pattern follows the rolling-hinge models^23^. Hydrothermal circulation is driven by the twin detachment faults from the deep heat source at the depth of 13 ± 2 km bsf.

It has been widely accepted that a heat source of magmatic origin is required for high temperature and high heat output hydrothermal activity^46^. The existence of such a melt zone in the deep lithospheric mantle could be possible, if the focused melt delivery from the neighboring cold areas to the mantle beneath the Dragon Horn area, as revealed by the recent 2D seismic observations, are considered^16^. With the existence of this focused melt, the temperature of the deep front of the DF2 could easily reach 650 °C to drive the hydrothermal circulation. Overall, we suggest that the heat source for the Longqi-1 high-temperature hydrothermal circulation would most likely be a melt zone in the lithospheric mantle at a depth of ~13 ± 2 km bsf, which is associated with the deeply seated large-scale multi-stage detachment faulting system (Fig. 4).

Combining insights from the microearthquake activity in the brittle lithosphere, the long fluids-rock reaction path revealed by the complex fluid chemistry and fault geometry, and the numerical modeling of hydrothermal circulation system, we suggest that the hydrothermal circulation along the ultra-slow spreading ridge may reach as deep as ~13 ± 2 km bsf. This is considerably greater than that of the TAG and Logatchev-1 hydrothermal vent fields at the MAR^9,10^, and approximately 6 km deeper than the seismic Moho boundary in this region (~7 km bsf^16^). This means that hydrothermal circulation would penetrate into the upper lithospheric mantle. These inferred deeper and longer hydrothermal circulation paths facilitate significant interaction between the seawater and the oceanic lithosphere, thereby affecting the thermal architecture and the composition of the oceanic lithosphere^22,50^ and the ore-forming capacity of the hydrothermal activity in ultra-slow spreading environments^6^.

## Methods

### Microearthquakes location

More than 9 months of passive seismic data were recorded using two kinds of free fall OBS with hydrophone: German Geopro Sedis IV (60 s–50 Hz) and Chinese 1–4C long-period OBS (30 s–50 Hz). These were deployed during the Chinese Cruises DY34^th^, DY40^th^, and DY43^th^. We manually picked 3885 P-phases on the hydrophone channel and 3232 S-phases on the horizontal channels with average picking uncertainties of 0.05 and 0.15 s, respectively, as the input data for 967 events. Hypocenters were located by the least-squares HYPOSAT routine^51^ on the basis of a one-dimensional P-wave velocity profile derived from the three-dimensional velocity structure of the crust and upper mantle from wide-angle reflection data^15^. Events with azimuthal station gaps less than 270°, a root-mean square (RMS) travel time residual less than 0.4 s, with at least three P-phases and one S-phase and a free depth inverted solution, were located as the initial hypocenters prior to relocation. The mean horizontal error semi-major and minor axis of the 95% confidence error ellipsoid are 2.19 and 1.79 km, respectively, with a depth error of 2.21 km. Finally, 512 well located events were relocated by the double-difference algorithm. In double-difference theory, the nearest-neighbor events over distance at common stations are much smaller than the length scale of the assumed velocity variation sampled by the seismic waves, and thereby model errors originating from outside the source area are reduced^52^. Compared with the events located initially by HYPOSAT, the average root-mean square travel-time residual for the relocated events decreased from 0.2 to 0.018 s.

### Sampling and analysis of hydrothermal vent fluids

Hydrothermal fluid samples from the sulfide structures at Longqi-1 vent area were collected using pressurized, piston-driven sampling devices, constructed entirely of titanium. These devices make use of compressed nitrogen to maintain each sample at seafloor pressure before and during sampling, and are similar in design and concept to IGT samplers developed in ref. ^53^. Immediately after arrival of the samplers on board, aliquots were taken for pH, and measurement of the dissolved gases (H_2_, H_2_S, CH_4_) were undertaken. For a detailed description of on-board analytical methods see refs. ^28,53^. Additional sub-samples were preserved for shore-based analysis of major dissolved anions/cations and trace metals. The trace metal aliquot was immediately acidified with analytical grade HCl (Optima). A fraction of this sub-sample was then diluted 50-fold and utilized for analysis of aqueous silica. Both fractions were stored in pre-weighed and acid cleaned high-density polyethylene bottles. Precipitates that formed in the titanium samplers were collected on a 0.45-μm nylon filter and subsequently re-dissolved in HCl/HNO_3_ (Ultrex). Based on the total volume of the particular sampler used, the amount of metals measured in the precipitate was recombined with metals that remained in solution to obtain a complete metal inventory for the vent fluid samples. Essentially, this process only affected Fe, Cu, and Zn. For vent fluid samples above 300 °C, the precipitates account for 90% of the reported concentrations of Cu and Zn, but less than 10% of the Fe^28^. The shore-based analysis of all major dissolved cations/anions was conducted using ICP-OES and/or ion chromatography, while trace metals were determined by ICP-MS. The analytical uncertainties (2*σ*) is ±2% for Cl (Ion chromatography), ±10% for H_2_, CH_4_ (gas chromatography–TCD) and H_2_S (CuCl_2_-precipitation/H_2_O_2_ reduction). Other elements are measured with ICP-MS, and 2*σ* uncertainties are ±2% for major species and ±5% for minor species^27^. Detection limits by ICP-MS are approximately an order of magnitude below reported concentrations. The hydrogen and oxygen isotopes of hydrothermal vent fluids were measured at the University of Arizona using a Thermo Finnigan Delta XP isotope ratio mass spectrometer. All samples were measured at least in duplicate and results are principally given in the standard delta notation in per mil (‰) vs. VSMOW according to δ^18^O (D) [‰] = (*R*_sample_/*R*_reference_−R_reference_) × 1000 fluid samples^54^. The endmember composition of hydrothermal fluids was calculated using a least-squares regression of the individual components versus Mg with extrapolation to Mg zero and including the respective seawater values. Vent fluid chemistry is summarized in Supplementary Tables 1 and 2.

### 2D numerical model

Based on the vent temperature and seismic data discussed above, we constructed a 2D numerical model for hydrothermal circulation along a profile across Longqi-1 field and the ridge axis (shown as AA′ in Fig. 1b). The governing equations for convection of seawater and thermodynamic properties calculated from equations in ref. ^55^, and are solved on an unstructured triangular mesh (see Supplementary Fig. 3) using a hydrothermal model solving for porous flow of water(see ref. ^47^ for details). The heat source is simulated by a fixed temperature boundary condition at the base. The detailed boundary conditions and mesh structure are shown in Supplementary Fig. 3.

No high-temperature vents and fewer discrete earthquakes are associated with DF1 and thereby, we inferred that DF1 might be inactive. So, here we only consider DF2 as the permeable zone in the 2D numerical model. The precise width of DF2 cannot be inferred from the seismic data, so we conducted a series of calculations with width (*d*) of DF2 varying from 100 to 1000 m. DF2 is considered as the main pathway of hydrothermal upwelling. There are no data to constrain the precise permeability distribution within DF2; however, the permeability of the discharge zone (*k*_d_) can be estimated from the heat output (*H*), vent temperature (*T*_d_ = 379 °C), and area (*A*_d_ = 8 × 10^4^ m^2^) of vent field based on the single pass model^56^,

1 $$Q = \frac{H}{{c_{\mathrm{{ht}}}T_{\mathrm{d}}}},$$

2 $$k_{\mathrm{d}} = \frac{{Qv_{\mathrm{d}}}}{{\rho _{f0}a_{\mathrm{d}}gT_{\mathrm{d}}A_{\mathrm{d}}}},$$

where *c*_ht_ ≈ 5 × 10^3^ J kg^−1^ °C^−1^, *α*_d_ ≈ 10^−3^ °C^−1^, and *ν*_d_ ~ 10^−7^ m^2^ s^−1^ are the specific heat, thermal expansion coefficient, kinematic viscosity of the high-temperature venting fluid, respectively. $\rho _{f_0}$ = 1000 kg m^−3^ is the density of fluid at 0 °C; *g* = 9.8 m s^−2^ is the gravitational acceleration. The total heat output of high-temperature focused flow is *H* = 250 ± 100 MW and is estimated by the heat output of hydrothermal plumes detected by the autonomous underwater vehicle data^45^. Substituting these parameters in Eqs. (1) and (2), we can obtain a mass flow rate of *Q* = 132 ± 52 kg s^−1^ and the permeability of the discharge zone *k*_d_ = 3 × 10^−14^ ∼ 6 × 10^−14^ m^2^, which is also used as the permeability of detachment fault *k*_df_. The background permeability *k*_b_ = 5 × 10^−16^ m^2^ is chosen such that a homogeneous model without detachment faults predicts high-temperature venting^24^.

## Supplementary information

Supplementary Information

## Data Availability

The geochemical data for the fluids used in this study are reported in the Supplementary Tables, and the seismic data may be requested from the corresponding author Chunhui Tao (taochunhuimail@163.com).

## Code availability

The readers can access the open source code on the website as follows:

1. HYPOSAT for seismic location (ftp://ftp.norsar.no/pub/outgoing/johannes/hyposat), for some details about program package see ref. ^51^

2. Numerical modeling codes are available upon request from the corresponding author Hasenclever et al. (2014).
