# Supplementary material for: Deep high-temperature hydrothermal circulation in a detachment faulting system on the ultra-slow spreading ridge
Source: Nat Commun. 2020 Mar 10;11:1300. doi: 10.1038/s41467-020-15062-w (PMC7064610; doi:10.1038/s41467-020-15062-w)
Supplement: Supplementary file 1 — Supplementary Information [file 41467_2020_15062_MOESM1_ESM.pdf]

Supplementary Information for

Deep high-temperature hydrothermal circulation in a detachment faulting system on the ultra-slow spreading ridge

Chunhui Tao et al.

List of Supplementary Figures

Supplementary Figure 1. The Dragon Horn area overview shows distribution of microearthquakes in horizontal plan.

Supplementary Figure 2. AA' seismic velocity profile across the ridge.

Supplementary Figure 3. The 2D numerical modeling detail.

Supplementary Figure 4. Temperature field and vent mass flux response to fault width and permeability contrast.

Supplementary Figure 5. Temperature field and mass flux response to DF1.

List of Supplementary Tables

Supplementary Table 1. End-member composition of vent fluids from the Longqi-1 field.

Supplementary Table 2. Oxygen and hydrogen isotope of vent fluids from the Longqi-1 field.

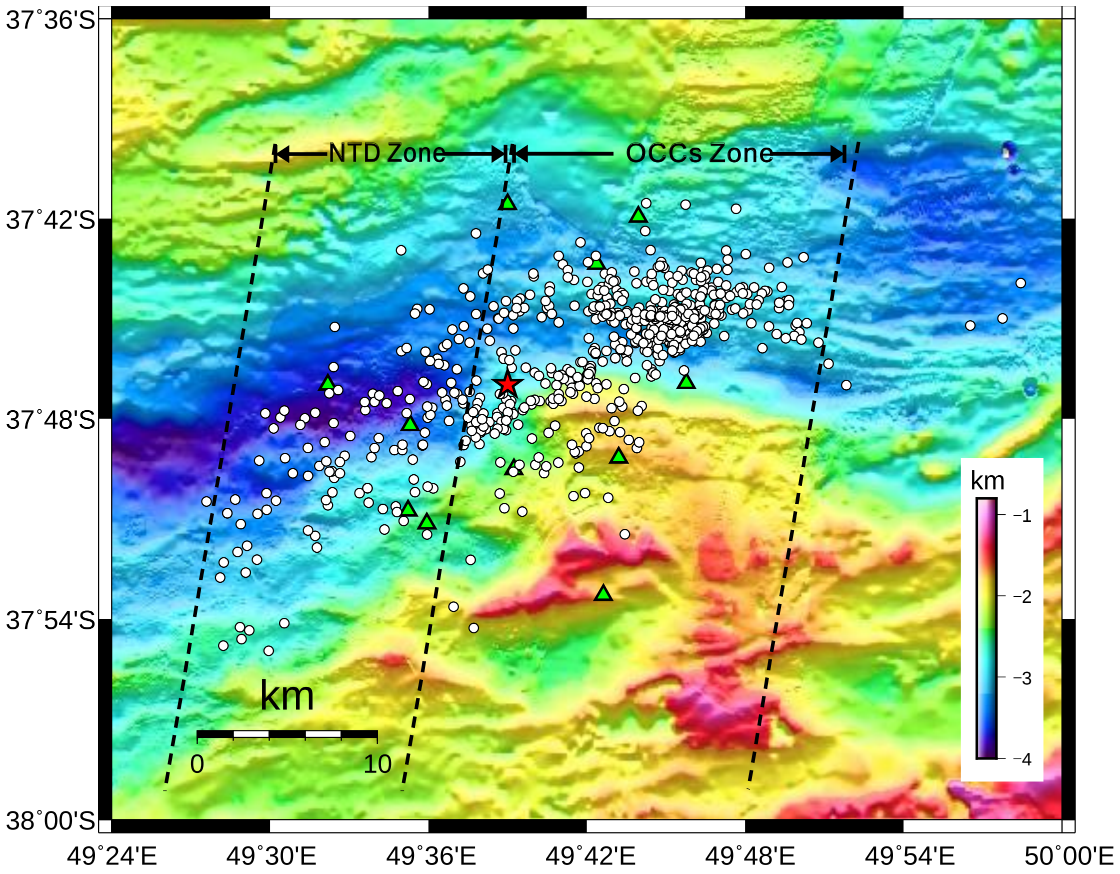

Supplementary Figure 1. The Dragon Horn area overview shows distribution of microearthquakes in horizontal plan. Earthquakes activities are divided into two sections: NTD and OCCs zone, and only epicenters located in OCCs zone are projected on the AA' profile in Fig. 1b. The green triangles show the location of OBSs. The active Longqi-1 field is shown as the red star.

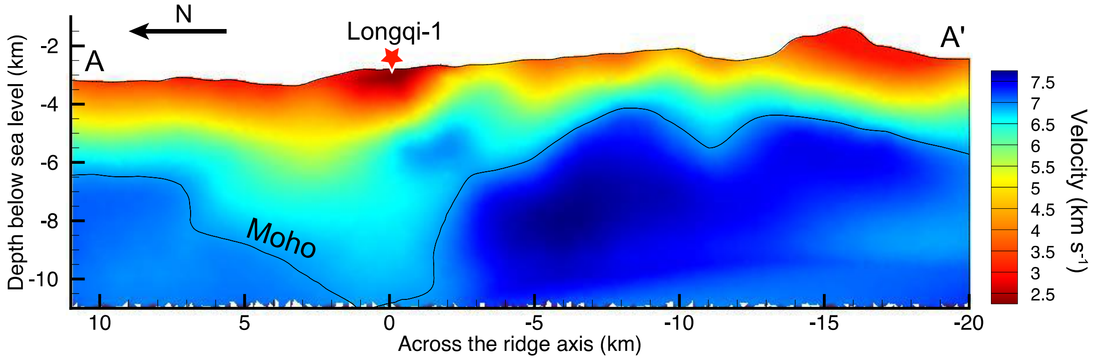

Supplementary Figure 2. AA′ seismic velocity profile across the ridge. Seismic data come from a wide-angle seismic experiment of ref[1].

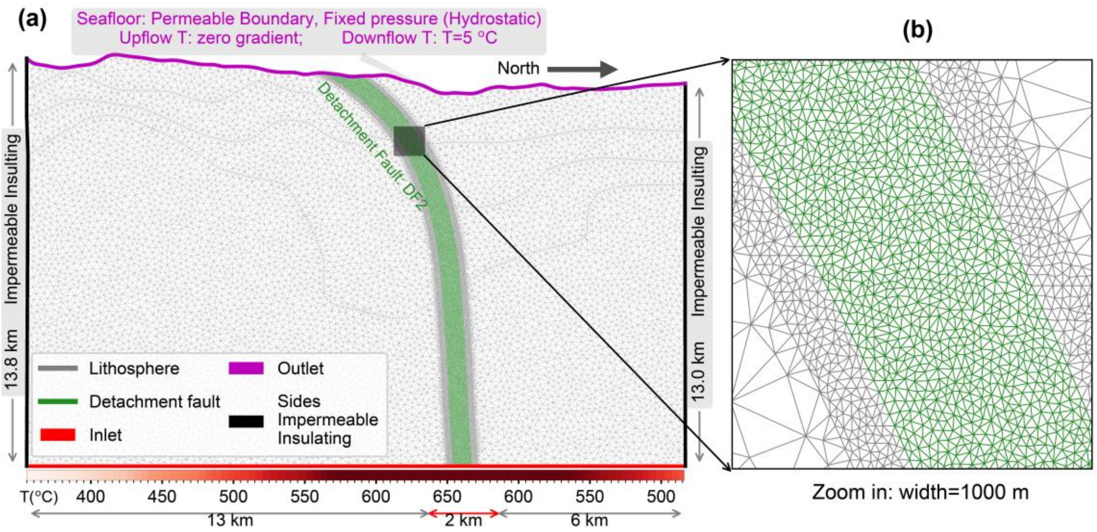

Supplementary Figure 3. The 2D numerical modeling detail. a. Illustration of the geometry, boundary conditions and permeability structure of the numerical model. The model region is discretized as triangle mesh. Detachment fault 2 with permeability k_{df2} and lithosphere with permeability k_b (background permeability) are shown in green and gray, respectively. Top boundary is the seafloor along the profile AA' in Fig. 1b. The pressure boundary condition at top is set to hydrostatic pressure according to bathymetry. Temperature boundary condition at top is a mixed boundary condition, which is set to zero gradient when fluid flows outward and set to fixed value (2°C) when fluid flows inward. Heat source at bottom boundary is set to fixed temperature of 650°C according to microearthquake data (Fig. 1c). Width of heat source is set to 2 km which is a similar to the Logitachev-1 model[2]. The temperature profile at bottom boundary is linearly decreasing from heat source (near detachment fault) to sides, which is similar to the numerical models by ref [3] and ref [4]. Two side boundaries are impermeable and insulting. **b.** The mesh structure details of the region within and near the fault.

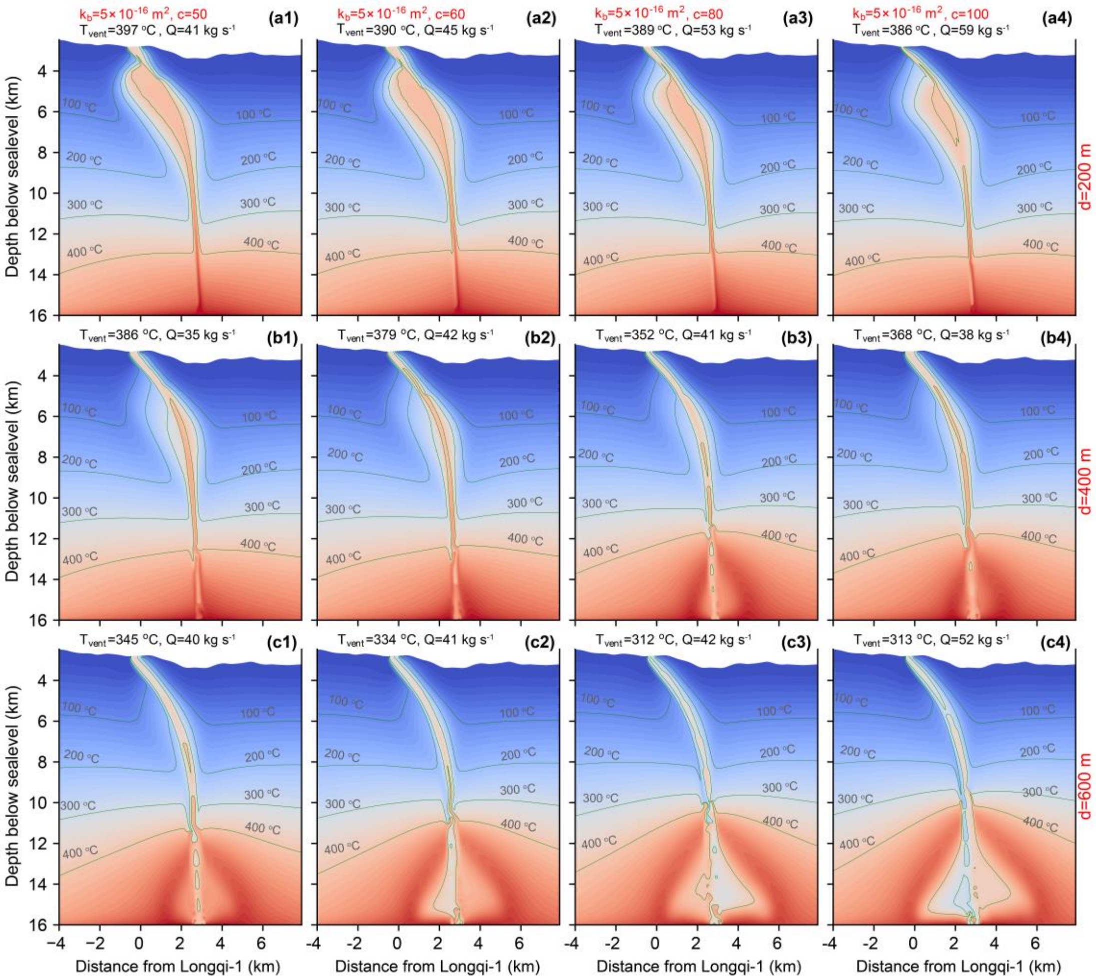

Supplementary Figure 4. Temperature field and vent mass flux response to fault width and permeability contrast. **a1-c4,** the specific modeling result with different inputs. Fault width d=200, 400, 600 m (each row: a, b, c) and permeability contrast c=k_{df2}/k_b=50, 60, 80, 100 (each column: 1, 2, 3, 4). The color scale of temperature field is the same as Fig. 3a in the main text.

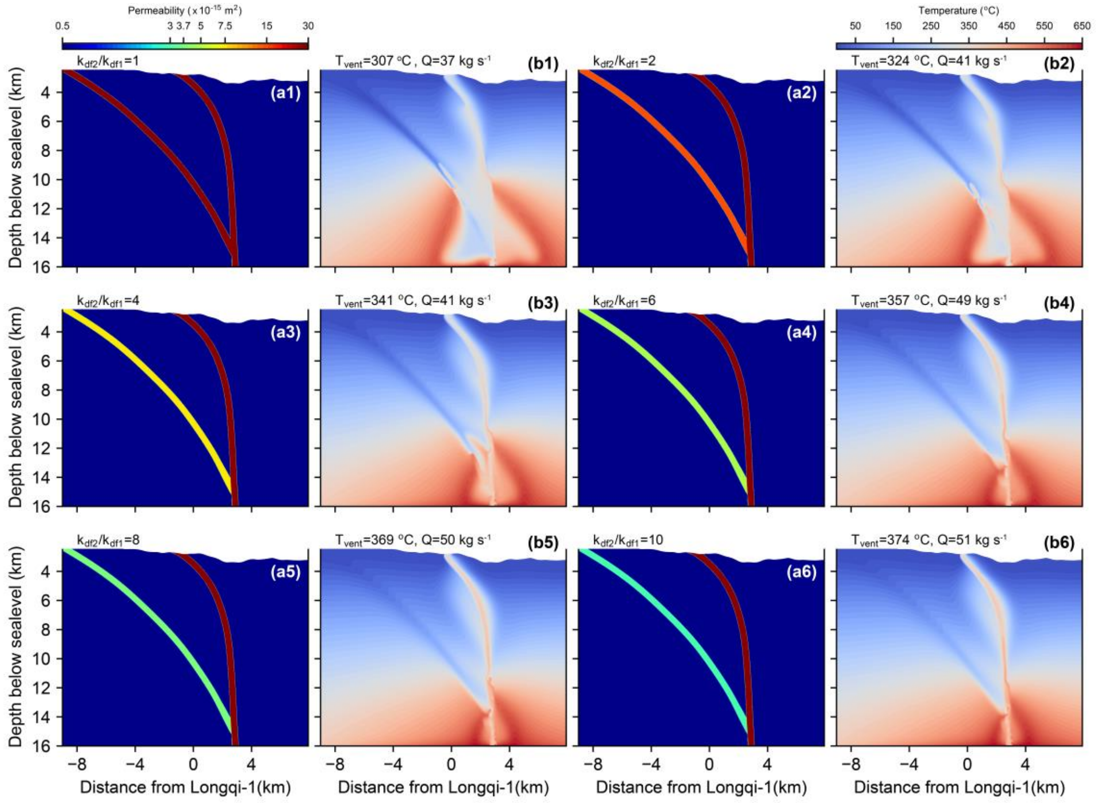

Supplementary Figure 5. Temperature field and mass flux response to DF1. Permeability distribution of each models are shown in odd columns (a1~a6), and temperature fields are shown in even columns (b1~b6). Color scale of permeability distribution of each model are the same, shown at the top of the subfigure (a1~a6). Color scale of temperature field is the same as Fig. 3 in the main text. The other parameters, such as d=400, k_b=5 × 10^{-16} m^2 and c=k_{df2}/k_b=60, are the same as the model of Fig 3a.

Supplementary Table 1. End-member composition of vent fluids from the Longqi-1 field.

Vent	Unit	**S Zone**			**M Zone**	TAG	Sea water
		**DFF3**	**DFF5**	**DFF20**	**DFF6**		
Max T	°C	352	146	362	379	366	2
Lowest pH**	-	3.21	4.85	3.32	3.42	3.35	7.44
Composition							
Min Mg	mmol kg^{-1}	4.88	25.7	6.04	1.86	2.84	52.8
H_2	mmol kg^{-1}	0.19	-	0.31	0.37	0.25	-
CH_4	mmol kg^{-1}	0.33	-	0.38	0.35	0.18	-
Cl	mmol kg^{-1}	594	718	605	596	636	545
H_2S	mmol kg^{-1}	4.7	1.8	5.9	6.9	3.5	-
Ca	mmol kg^{-1}	45	50	42	45	30	10.28
K	mmol kg^{-1}	14.7	17	14	14	17.1	10.21
Na	mmol kg^{-1}	-	540	463	471	543	464
Fe	mmol kg^{-1}	12	9	11.3	13.7	5.6	0.01
Mn	mmol kg^{-1}	1.80	2.1	1.60	1.65	0.68	0.01
Si	µmol kg^{-1}	17	17	16	17	20.7	0.1
Sr	µmol kg^{-1}	95	93	93	96	99	90
Li	µmol kg^{-1}	730	825	680	710	368	24
B	µmol kg^{-1}	-	375	376	365	365	416
Rb	µmol kg^{-1}	10.5	12.5	9	10.1	9.1	12
Cs	µmol kg^{-1}	0.085	0.135	0.099	0.110	0.108	0.0022
Cu***	µmol kg^{-1}	21	-	42	9	150	0.01
Zn***	µmol kg^{-1}	303	-	-	327	46	0.01

Note: Endmember composition calculated as discussed in ref[5] and methods section.

-: not determined.

**: pH measured at 25 ° C and 1 atm

***: total Cu and Zn including dissolved phase and dregs[5]

t: TAG data from the following sources [6,7,8]

The analytical uncertainties (2σ) is ±2% for Cl (Ion chromatography), ±10% for H_2, CH_4 (Gas chromatography—TCD) and H_2S ($CuCl_2$-precipitation/H_2O_2 reduction). Other elements are measured with ICP-MS, and 2σ uncertainties are ±2% for major species, ±5% for minor species (ref[5]).

Supplementary Table 2. Oxygen and hydrogen isotope of vent fluids from the Longqi-1 field.

Vent	Dive-sampler	$\delta^{18}O$	$\Delta^{18}O$	δD	ΔD
	Unit	‰	‰	‰	‰
DFF3	JL 89-CGT-B	1.21	1.48	3.4	4.6
DFF3	JL 89-CGT-C	0.18		0.8	
DFF6	JL 95-CGT-C	1.21	1.48	3.9	5.1
DFF20	JL 96-CGT-D	1.14	1.14	3.8	5.0
DFF20	JL 96-CGT-E	0.65		2.3	
Bottom water		-0.27		-1.2	

Note: See ref [10] for method; The Values of $\delta^{18}O$ and δD are calibrated relative to the VSMOW standard and have reproducibility of approximately 0.1 and 1.0‰, respectively.

References

1. Zhao, M. et al. Three-dimensional seismic structure of the Dragon Flag oceanic core complex at the ultraslow spreading Southwest Indian Ridge (49° 39′ E). *Geochem. Geophy. Geosy.* **14**, 4544-4563 (2013).
2. Andersen, C., Rüpke, L., Hasenclever, J., Grevemeyer, I. & Petersen, S. Fault geometry and permeability contrast control vent temperatures at the Logatchev 1 hydrothermal field, Mid-Atlantic Ridge. *Geology* **43**, 51-54 (2015).
3. Lowell, R., Gosnell, S. & Yang, Y. Numerical simulations of single‐pass hydrothermal convection at mid‐ocean ridges: Effects of the extrusive layer and temperature‐dependent permeability. *Geochem. Geophy. Geosy.* **8**,10 (2007).
4. Lewis, K. & Lowell, R. Numerical modeling of two‐phase flow in the NaCl‐H2O system: Introduction of a numerical method and benchmarking. *J. Geophys. Res.: Sol. Ea.* **114**, B5 (2009).
5. Seyfried Jr, W. E., Foustoukos, D. I., & Fu, Q. (2007). Redox evolution and mass transfer during serpentinization: An experimental and theoretical study at 200 C, 500 bar with implications for ultramafic-hosted hydrothermal systems at Mid-Ocean Ridges. Geochimica et Cosmochimica Acta, 71(15), 3872-3886.
6. Charlou, J.L., Donval, J.P., Jean-Baptiste, P., Dapoigny, A. & Rona, P.A. Gases and helium isotopes in high temperature solutions sampled before and after ODP Leg 158 drilling at TAG Hydrothermal Field (26°N, MAR). *Geophys. Res. Lett.* **23**, 3491-3494 (1996).
7. Edmond, J.M., Campbell, A.C., Palmer, M.R., Klinkhammer, G.P., German, C.R., Edmonds, H.N., Elderfield, H., Thompson, G. and Rona, P. Time series studies of vent fluids from the TAG and MARK sites (1986, 1990) Mid-Atlantic Ridge: a new solution chemistry model and a mechanism for Cu/Zn zonation in massive sulphide orebodies: *Geological Society, London, Special Publications* **87**, 77-86 (1995).

8. Edmonds, H.N. et al. Continuation of the hydrothermal fluid chemistry time series at TAG, and the effects of ODP drilling. *Geophys. Res. Lett.* **23**, 3487-3489 (1996).
9. Seewald, J., Cruse, A., & Saccocia, P. Aqueous volatiles in hydrothermal fluids from the Main Endeavour Field, northern Juan de Fuca Ridge: temporal variability following earthquake activity. *Earth Planet. Sci. Lett.*, **216**, 575-590 (2003).
10. Shanks, W.C. Stable Isotopes in Seafloor Hydrothermal Systems: Vent fluids, hydrothermal deposits, hydrothermal alteration, and microbial processes: *Rev. Mineral. Geochem*. **43**, 469-525 (2001).
